# Continuous Curvilinear Capsulorhexis in Cataract Surgery Using a Modified 3-Bend Cystotome

**DOI:** 10.1155/2015/412810

**Published:** 2015-10-05

**Authors:** Yuan Zeng, Jian-hua Gao

**Affiliations:** Department of Ophthalmology, Kunming General Hospital of Chengdu Military Command, Kunming, Yunnan 650032, China

## Abstract

We modified a 2-bend cystotome for continuous curvilinear capsulorhexis (CCC) in manual or phacoemulsification cataract surgery to improve the safety and ease of performance. A 26G needle was converted into a cystotome with 3 bends. In this retrospective study, the performance of modified 3-bend cystotome was compared with conventional 2-bend cystotome. During cataract surgery, in the 3-bend cystotome group, mean completion time of CCC was shorter, mean times of viscoelastic agent supplement were less, and CCC success rate was higher than that in 2-bend group. Complication incidence, such as postoperative transient corneal edema and irreparable V-shaped tear, was also lower in 3-bend group. No posterior capsular rupture or no other complication was observed in either group. A polymethyl methacrylate intraocular lens or a hydrogel intraocular lens was implanted in the capsular bag in all eyes. We conclude that it is safe and efficient to accomplish a CCC using the 3-bend cystotome due to its ability to sustain the anterior chamber depth (ACD) and keep the posterior lip intact. Using the 3-bend cystotome also allowed for an adequate view into the anterior chamber from lack of wound deformation.

## 1. Introduction

Continuous curvilinear capsulorhexis (CCC) is considered the standard and a critical step of anterior capsule opening in modern cataract surgery (either phacoemulsification or manual sutureless extracapsular cataract extraction) [1]. Cystotome and forceps are two of the most commonly used instruments for a CCC, despite the rise of femtosecond laser assisted capsulorhexis [2–4]. According to previously published results in a porcine eye model, femtosecond laser assisted capsulorhexis had less average resistance to capsule tear than CCC because the edge of the anterior capsule opening made by femtosecond laser was not as smooth as that made manually [5]. The advantages of using a cystotome to create a capsulorhexis compared to a pair of forceps include less corneal wound distortion, better view of the capsulorhexis edge, and minimal inadvertent loss of the viscoelastic agent. In addition, the cystotome can be mounted to a syringe containing the viscoelastic agent, which facilitates viscoelastic agent supplementation if necessary [6]. A cystotome is also cheaper than a pair of forceps.

A conventional cystotome has 2 bends, which may not keep the depth of the anterior chamber stable and therefore result in CCC failure. In the present study, we describe the workflow for a CCC using a modified 3-bend cystotome instrument and compare our results to those obtained by using a 2-bend cystotome.

## 2. Materials and Methods

The present study adhered to the tenets of the Declaration of Helsinki and was approved by the local ethics committee. Informed consent for retrospective data analysis was obtained from cataract surgery candidates after explanation of the nature and possible consequences of the study, and approval from the local ethics committee (number 2006019) was obtained.

### 2.1. Subjects

The medical records of all cases with a cataract diagnosis were retrieved from the Department of Ophthalmology at the number 535 Hospital of Chinese PLA between June 2009 and January 2010 and reviewed. One hundred and eighty-four patients covering 295 eyes were included in our study. All the cases were performed by a single surgeon (Deng J-w). One hundred and forty-two consecutive eyes using a 2-bend cystotome from June to October 2009 were enrolled in the 2-bend group (control group), and one hundred and fifty-three consecutive eyes from November 2009 to January 2010 using the 3 bend-cystotome technique were enrolled in the 3-bend group (study group). The cataract surgeries in the present study were performed using manual sutureless extracapsular cataract extraction (ECCE) or phacoemulsification. In the 3-bend group, 127 eyes were operated on using manual sutureless ECCE and 26 eyes, using phacoemulsification. In the 2-bend group, 117 eyes were operated on using manual sutureless ECCE and 25 eyes, using phacoemulsification.

Exclusion criteria were pediatric cataracts, anterior capsule calcification, posterior synechia of the iris, and hypermature cataract cases with a liquefied cortex.

Table 1 shows the preoperative information of the two groups. The two groups were comparable with respect to age, female ratio, nucleus hardness, and ECCE/PE ratio (*P* = 0.09, 0.94, 0.40, and 0.78, resp.).

### 2.2. Techniques of Making and Using a 3-Bend Cystotome

**Figure 1:**
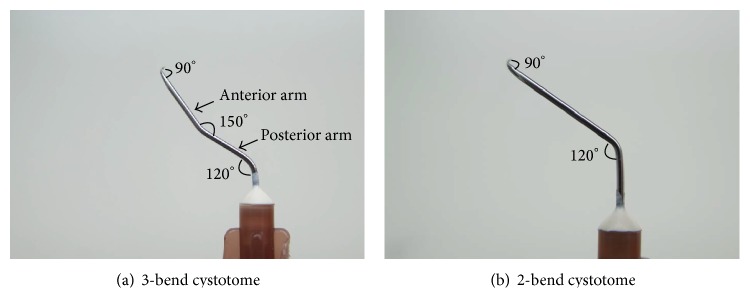
Demonstration of 2- and 3-bend cystotomes. Compared to the 2-bend cystotome, the 3-bend cystotome has one more bend at the middle point of the syringe needle. The three bends are 90° at the bevel, 120° at the hub, and 150° at the middle point.

Using a microneedle holder, three-bend cystotome is converted from a disposable 26G needle with a sharp tip. Compared to the conventional 2-bend cystotome, a 3-bend cystotome has one more bend at the middle point of the syringe needle. The three bends are 90° at the bevel, 120° at the hub, and 150° at the middle point (Figure 1). The angle at the middle point can also be adjusted according to the anterior chamber depth (ACD). For instance, the surgeon can bend the needle more (140°) if the ACD is deeper or less (160°) if the ACD is shallower.

The CCC procedure when using a 3-bend cystotome is the same as a routine CCC. Performing a typical CCC includes four steps: the initial cut, raising a flap, tearing the flap, and completing the rhexis. Capsulorhexis is made in either a clockwise or counterclockwise direction. The flap is torn either by ripping force or shearing force.

### 2.3. Cataract Surgery Procedure

#### 2.3.1. Manual Sutureless Extracapsular Cataract Extraction

A fornix-based conjunctival flap was made under topical anesthesia (oxybuprocaine eye drops). A 7.0 mm to 8.0 mm straight scleral incision 1.5 mm from the limbus was marked with calipers on the surface of the sclera, avoiding the major scleral vessels. A superficial scleral tunnel was dissected to the clear cornea using a crescent scalpel. The anterior chamber was entered via the clear cornea using a keratome. Paracentesis was made at the 10:00 position using a stiletto knife. A side port entry site was made at the 9:00 position. The anterior chamber was filled with viscoelastic material and a 7.0 mm diameter capsulorhexis was initiated using a cystotome. Once capsulorhexis was completed, the wound was enlarged internally to 9.0–10.0 mm according to the size of the nucleus.

After hydrodissection of the nucleus using a filtered balanced saline solution, a Sinskey hook was embedded in the nucleus and pushed toward the 7:00 position. Once the superior pole of the nucleus was visualized, a Kuglen hook was inserted underneath. Both instruments were passed through the main wound, and the pole was then tipped up with the Kuglen hook. The nucleus was removed from the capsular bag by alternately engaging the equator with the Sinskey hook and Kuglen hook.

To perform the nuclear extraction, the Sinskey hook was held in the right hand and the Kuglen hook was held in the left hand. The Sinskey hook was inserted into the anterior chamber between the nucleus and the cornea; the tip of the hook was then embedded into the centre of the nucleus. The main wound was then opened to a fish-mouth shape by lifting the end of the Sinskey hook. Two hands were used simultaneously, with the right hand pulling the nucleus and the left hand pressing on the scleral bed 2 mm behind the posterior flap. Increased intraocular pressure and the pulling force exerted by the Sinskey hook dislodged the nucleus from the eyeball in its entirety without fragmentation in the anterior chamber or tunnel. The force on the scleral bed was exerted continuously and slowly. Throughout the procedure, special care was taken not to grasp the iris or capsule.

The residual epinucleus was hydroexpressed using a Simcoe cannula through the scleral incision. After cortex aspiration, a polymethyl methacrylate intraocular lens (IOL) was implanted in the capsular bag and the incision verified to ensure it had self-sealed. No suture was placed.

#### 2.3.2. Clear Corneal Tunnel Phacoemulsification

Seventy-five percent of the corneal thickness was calculated and used to set the depth of the incision using a diamond knife. A stab incision was made at the left side of the incision and the chamber was filled with viscoelastic material. A 3.0 mm keratome blade was inserted into the lamellar wound dissection. The three-plane incision was completed by pointing the tip of the keratome toward the lens and slowly inserting the blade to its full extent to produce a square 3.0 mm tunnel. After continuous curvilinear capsulorhexis and nucleus hydrodissection, phacoemulsification was performed. Injector cartridge systems were used to inject the posterior chamber IOLs. The viscoelastic agent was removed using the irrigation aspiration handpiece, and balanced salt solution was injected through the paracentesis tract to deepen the anterior chamber.

### 2.4. Statistical Analysis

Student's two-tailed *t*-tests were used to compare measurement data between the two groups. Pearson's chi-square test was used to compare percentages between the two groups. *P* < 0.05 was considered statistically significant.

## 3. Results

During cataract surgery, mean CCC completion time was 5.6 ± 2.9 seconds in the 3-bend group and 8.9 ± 4.5 seconds in the 2-bend group (*P* < 0.001). The mean number of times for the viscoelastic agent supplement was 0.3 ± 0.2 and 1.4 ± 0.6 in the 3-bend group and 2-bend group, respectively (*P* < 0.001). CCC was completed successfully in 151 eyes (98.7%) for the 3-bend group, whereas the success rate in the 2-bend group was 90.1% (*P* = 0.001). Postoperative transient corneal edema was noted in 2 eyes and 14 eyes in the 3-bend and 2-bend groups, respectively (*P* = 0.002). The rate of best corrected visual acuity better than 5/10 was comparable between both groups 1 day and 3 months following surgery (*P* = 0.24 and 0.89, resp.) (Table 2).

A V-shaped tear remained after a peripheral radial tear-out noted in 2 eyes (1.3%) of the 3-bend group and 14 eyes (9.9%) of the 2-bend group. The anterior capsule opening was performed from the opposite direction when such a radial tear occurred. The remaining procedure was finished without further complication. No posterior capsular rupture or other complications were observed in either group. A polymethyl methacrylate or hydrogel intraocular lens was implanted in the capsular bag in all eyes.

## 4. Discussion

The importance of a perfect CCC for a cataract surgery cannot be overemphasized. Sustaining an adequate ACD is the prerequisite of performing a CCC successfully. If the anterior chamber becomes shallow and an ophthalmic viscoelastic agent is not added in a timely manner, pressure from the vitreous body will push the lens upward, which increases zonular tension. Consequently, there is a higher risk for the capsular flap to tear at the periphery. Once the capsular flap becomes uncontrollable, it can extend around the equator into the posterior capsule, compromising the integrity of the capsular bag. Resulting consequences include vitreous loss, a residual nucleus or cortex, abortion of the intraocular lens implantation, and suboptimal intraocular lens location and stability [7–12], all of which can be major medical issues.

Ernest introduced the concept of the posterior corneal lip to prevent fluid escape from the anterior chamber [13]. This lip was also intended to prevent hyphema, which occurred in 5% to 10% of the patients undergoing scleral tunnel incisions. The posterior corneal lip soon proved to be more important than the long scleral tunnel with vertical cuts in the tunnel floor. The three-step procedure leaves an internal lip, which comprises endothelium, Descemet's membrane, and corneal stroma. The internal lip seals on itself when the intraocular pressure returns to normal. In cadaver eye studies, the posterior corneal lip incision produced a wound that restricted leakage and iris prolapse at hydrostatic pressure exceeding 400 mmHg. The posterior lip is also the key device in preventing the viscoelastic agent running off from the anterior chamber. Thus, to keep a stable ACD, the posterior lip should not be stressed. However, trainee cataract surgeons often press the posterior lip unintentionally when they only focus on the capsular flap being processed. This common mistake that a trainee cataract surgeon makes would be more severe when using a conventional 2-bend cystotome.

The conventional 2-bend cystotome has only one straight arm, which inevitably presses the posterior lip when performing a CCC, allowing the viscoelastic agent to escape and failing to sustain the ACD. A 3-bend cystotome is similar to a human arm, with the middle bend mimicking the elbow. The posterior arm conforms to the angle of the posterior lip, and the ACD is still kept stable when the anterior arm drives the tip to perform a CCC.

**Figure 2:**
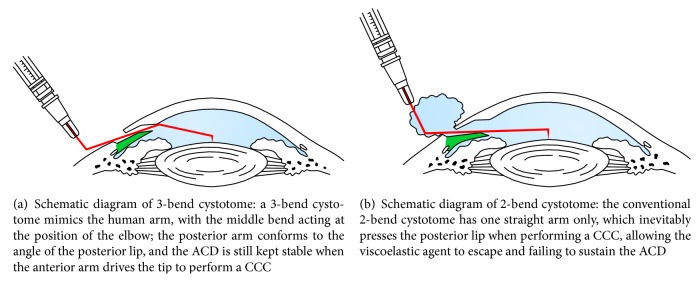
Schematic diagram of 3- and 2-bend cystotomes.

Perfect visualization is of great importance when performing a CCC. The 2-bend cystotome produces wrinkles around the corneal incision when pressing the posterior lip, obscuring any observation of flap tearing. In contrast, a 3-bend cystotome does not stress the posterior lip, so that there is no distortion of the cornea and clear visualization is maintained (Figure 2).

The present study suggests that performing CCC in a cataract surgery using the 3-bend cystotome leads to a higher success rate with less surgical time when compared to surgery using a 2-bend cystotome.

The drawback of the 3-bend cystotome, like the 2-bend cystotome, is that it must be made each time before a single surgery by converting a 26G needle. This can be time consuming when compared with using capsulorhexis forceps. In hypermature cataracts with a liquefied cortex, the forceps technique is still preferred because a needle will not find the necessary counter pressure for engagement of the capsule. However, a cystotome is preferred for CCC in most cases, as forceps occupy more space and will not sustain a stable ACD.

In conclusion, a 3-bend cystotome is economical and allows for a safer and more efficient CCC procedure.

## Figures and Tables

**Table 1:** Characteristics of eyes in the 3-bend and 2-bend groups before surgery.

Demographics	3-bend cystotome	2-bend cystotome	*P* value
	(*n* = 153)	(*n* = 142)	
Age (year)	74.3 ± 5.8	73.1 ± 6.5	0.09
Female ratio	51%	51.4%	0.94
Nucleus hardness^*∗*^	4.16 ± 0.81	4.25 ± 1.03	0.40
ECCE/PE ratio^#^	4.88	4.68	0.78

^*∗*^Emery-Little classification yielded the degrees of nucleus hardness.

^#^ECCE = extracapsular cataract extraction; PE = phacoemulsification.

**Table 2:** Characteristics of eyes in the 3-bend and 2-bend groups during and after surgery.

Demographics	3-bend cystotome (*n* = 153)	2-bend cystotome (*n* = 142)	*P* value
CT of CCC (second)	5.6 ± 2.9	8.9 ± 4.5	<0.001
Times of VAS	0.3 ± 0.2	1.4 ± 0.6	<0.001
CCC success rate (%)	98.7%	90.1%	0.001
CE incidence (%)	1.3%	9.2%	0.002
PBCVA ⩾ 5/10 (1 d)	76.5%	70.4%	0.24
PBCVA ⩾ 5/10 (3 m)	88.2%	88.7%	0.89

CT of CCC = completion time of continuous curvilinear capsulorhexis; times of VAS = times of viscoelastic agent supplement; CE = corneal edema; PBCVA = postoperative best corrected visual acuity.
